# Supplementary material for: Sex differences in wrist strength: a systematic review
Source: PeerJ. 2023 Dec 14;11:e16557. doi: 10.7717/peerj.16557 (PMC10725665; doi:10.7717/peerj.16557)
Supplement: Supplemental Information 2 [file peerj-11-16557-s002.docx]

November 4, 2023

Systematic Reviews and Meta-Analyses

We authors conducted this systematic review as there is a large body of research examining sex differences in wrist strength, but the interpretation of this data is hampered by the large variety in measurement techniques and participant populations. As ergonomic guidelines are often driven based on a singular or small number of former research studies, it is essential to properly collate and group this research to aid in the understanding of these differences. The purpose of this review was to assess the current existing literature on sex differences in wrist strength and to combine these findings to quantify changes in strength-generating abilities.
